# Acute coronary ischemia during alcohol withdrawal: a case report

**DOI:** 10.1186/1752-1947-5-369

**Published:** 2011-08-12

**Authors:** Chaturaka Rodrigo, Dhanesha Seneviratne Epa, Ganeshalingam Sriram, Saroj Jayasinghe

**Affiliations:** 1 University Medical Unit, National Hospital of Sri Lanka, Colombo, Sri Lanka; 2 Department of Clinical Medicine, Faculty of Medicine, University of Colombo, Sri Lanka

## Abstract

**Introduction:**

The potential of alcohol withdrawal to cause acute coronary events is an area that needs the urgent attention of clinicians and researchers.

**Case presentation:**

We report the case of a 52-year-old heavy-alcohol-using Sri Lankan man who developed electocardiogram changes suggestive of an acute coronary event during alcohol withdrawal. Despite the patient being asymptomatic, subsequent echocardiogram showed evidence of ischemic myocardial dysfunction. We review the literature on precipitation of myocardial ischemia during alcohol withdrawal and propose possible mechanisms.

**Conclusions:**

Alcohol withdrawal is a commonly observed phenomenon in hospitals. However, the number of cases reported in the literature of acute coronary events occurring during withdrawal is few. Many cases of acute ischemia or sudden cardiac deaths may be attributed to other well known complications of delirium tremens. This is an area needing the urgent attention of clinicians and epidemiologists.

## Introduction

The state of alcohol withdrawal is known for its life threatening complications such as delirium tremens. Several authors have observed the potential for it to cause acute coronary events [1,2], while others have observed subtle electrocardiogram (ECG) changes in patients during alcohol withdrawal [3]. We caution that this ominous complication should be expected and observed for while managing patients in alcohol withdrawal. We report the case of a man with acute coronary ischemia during alcohol withdrawal while under our care.

## Case presentation

A 52-year-old Sri Lankan man was transferred to the University Medical Unit (UMU) at the National Hospital of Sri Lanka, Colombo, for management of alcohol withdrawal. He had been a habitual heavy drinker with a daily consumption that was approximately 12 to 24 units of alcohol (as arrack, a locally brewed alcoholic beverage). His pattern of consumption had features of alcohol dependency such as tolerance, use despite knowing its harm, withdrawal features, neglect of alternate pleasures and unsuccessful efforts to cut down on usage.

On the day of admission, he had an episode of transient loss of consciousness with a fall and suffered a cut injury to his face. He was admitted to a surgical ward for wound care but developed features of alcohol withdrawal 48 hours after admission and was transferred to the UMU for further management.

He was restless and disoriented in time, place and person. There was a deep laceration over the left ear that was sutured. There were no clinical signs suggestive of hepatic or Wernicke's encephalopathy. He was managed with sedation, oral chlordiazepoxide, intravenous thiamin and adequate hydration. He did not develop seizures or fever during his stay in the hospital, and made a complete clinical recovery from the state of confusion within 72 hours.

**Figure 1:**
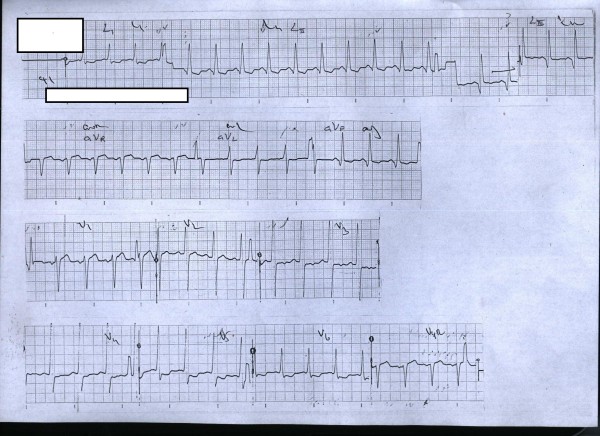
**The ECG on day 4**. ST segment depressions are visible in leads L1, L2, V5 and V6.

**Figure 2:**
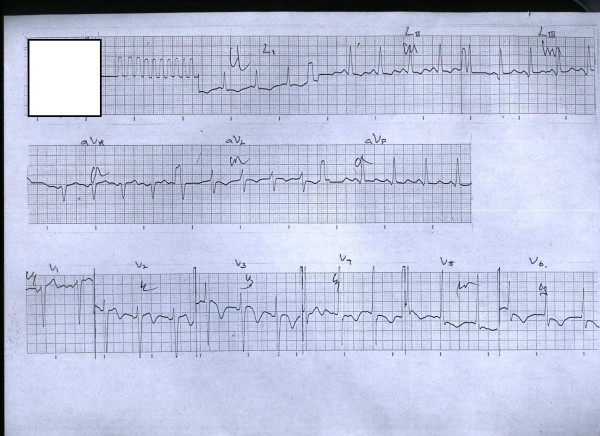
**The ECG on day 6**. There are additional changes of deep T inversions in leads aVL, V2-V6.

The ECG on admission was essentially normal and did not show abnormalities of ischemic heart disease. However, an ECG on day four (since admission) showed ST segment depressions in leads L1, L2, V5 and V6 (see Figure 1). The ECG on day five showed similar changes but they had progressed to significant (more than 2 mm) ST segment depression. The ECG on day six showed additional changes of deep T inversions in aVL and in precordial leads V2-V6 (see Figure 2). Despite not having typical chest pain, he was anticoagulated with low molecular weight heparin (enoxaparin) and was managed as for an acute coronary event (non invasive treatment strategy).

By this time, he had recovered from his delirium and was able to give a full history to assess his cardiovascular risk status. He had not had any acute coronary events in the past or any significant co-morbidity such as diabetes, hypertension or hypercholesterolemia. There was no significant family history but he was a heavy smoker (15 pack-years).

He had undetectable levels of Troponin I (sensitivity and specificity of approximately 90% at a cut-off of 0.5 ng/ml) on day six since admission. His liver enzyme levels in serum were elevated (ALT: 138 u/l, AST: 236 u/l). Serum sodium, potassium and creatinine were within the normal range. His hemoglobin level was 11.3 g/dl. There was no evidence of subdural hemorrhage on computed tomography (CT) scan which is an alternative cause for confusion and ECG changes. A subsequent echocardiogram showed septal and apical hypokinesia with evidence of ischemic left ventricular dysfunction.

He made a full recovery and was discharged on day ten with clinic follow up arranged. Since he was willing to abstain from alcohol, he was referred to counseling services at the University Psychiatry Unit.

## Discussion

Our patient showed ECG features of acute coronary ischemia during alcohol withdrawal. Though these could be mere coincidental events, there is growing evidence that supports alcohol withdrawal as a precipitant of acute coronary events. An accepted hypothesis is centered on the adrenergic surge occurring at the time of withdrawal [4]. The adrenergic stimulation to coronaries has a twofold action in the normal physiologic state: direct coronary vasoconstriction via α receptors and secondary coronary vasodilation via β receptors on the myocardium. Vasoconstriction occurring through α receptors (cutting down the coronary flow) is only transient. The β receptor stimulation increases the contractility of the myocardium which in turn increases the production of vasodilatory metabolites. This causes a secondary dilation of coronary vessels leading to a net improvement in flow. Perivascular fibrosis and intra-myocardial artery sclerosis that can potentially cause small vessel disease that limits the ability of the vessels to dilate at the time of an adrenergic crisis have been demonstrated in alcoholics [5]. This may precipitate an acute coronary event in a susceptible heart that is already damaged by long term alcohol use. Other theories suggest that magnesium deficiency and autonomic neuropathy (observed to occur with chronic alcoholism) derail the regulation of coronary vessels at a time of adrenergic crises which can precipitate an obstruction to flow [6,7].

The cause for the initial loss of consciousness and fall in this man is worth exploring. One possible explanation is that a transient arrhythmia precipitated the fall. Recent animal studies have shown that there is an imbalance between cardiac sympathetic and parasympathetic drive towards sympathetic predominance that potentially increases the risk for fatal arrhythmias during alcohol withdrawal. The degree of imbalance correlates with the non-homogeneity of cardiac repolarization [8,9]. These studies have also demonstrated a potential place for beta blocker pretreatment in reducing the repolarization abnormalities. In a case control study of human subjects Bar *et al*. have demonstrated that the QT interval is significantly prolonged in patients in acute alcohol withdrawal increasing the repolarization vulnerability of the myocardium. Authors assume that this prolongation is related to the sympathetic over activity during withdrawal [10]. The phenomenon of QT interval prolongation during alcohol withdrawal has also been investigated by Cuculi *et al*. [11]. They showed that in a sample of 49 patients with alcohol withdrawal, the majority (63%) had significant QT interval prolongation on ECG. The types of arrhythmias observed in this retrospective analysis included torsade de pointes, sustained ventricular tachycardia, atrial fibrillation and supraventricular tachycardia. Several others have also reported instances of QT interval prolongation in alcohol withdrawal including a case report of a neonate of an alcohol dependent mother developing QT interval prolongation and ventricular tachycardia after birth [12,13]. In addition to sympathetic over activity, there are many other contributory factors that may cause QT interval prolongation in a patient in alcohol withdrawal such as electrolyte disturbances, concurrent use of neuroleptics (for purposes of sedation) and renal and/or hepatic dysfunction. Although QT interval prolongation was not observed in our patient after hospital admission, the possibility of a transient arrhythmia precipitating the initial fall cannot be excluded.

While there are many plausible theories for vulnerability to acute coronary syndromes during alcohol withdrawal, clinical evidence for such an association is limited. Denison *et al*. [3] report ST segment changes in a case series of 19 men being treated for alcohol withdrawal. Seven patients in this case series had significant horizontal or down-sloping ST segment changes without any chest pain. Our patient did not have biochemical evidence of myocardial injury but Danenberg *et al*. reports a case in which a previously healthy individual had developed myocardial infarction during alcohol withdrawal [2]. There are only a few other reported cases where acute alcohol withdrawal is linked to acute coronary events and sudden cardiac death [1,14,15]. We have searched PUBMED with key words 'delirium tremens' or 'alcohol withdrawal' with 'acute coronary syndrome' appearing anywhere in the article and repeated the same search in Google Scholar (there were no time limits to the search). While acute coronary ischemia is a likely sequelae of alcohol withdrawal, given the observation of QT interval prolongation and arrhythmias in the studies quoted above, the significance of arrhythmias as a cause of sudden cardiac deaths must be considered as well.

## Conclusions

Given the fact that alcohol withdrawal is a commonly observed phenomenon in hospitals and the potential vulnerability to sudden cardiac death during withdrawal, the number of cases reported in the literature is few. It brings forth the question whether clinicians are actively observing for this potentially lethal complication of acute alcohol withdrawal. Many cases of acute ischemia or sudden cardiac deaths may go unnoticed and be attributed to other well-known complications of delirium tremens. This is an area that needs the urgent attention of researchers, epidemiologists and clinicians to establish the impact of acute alcohol withdrawal on cardiac morbidity and mortality.

## Consent

Written informed consent was obtained from the patient for publication of this case report and any accompanying images. A copy of the written consent is available for review by the Editor-in-Chief of this journal.

## Competing interests

The authors declare that they have no competing interests.

## Authors' contributions

All authors participated in designing, article search, information coding and writing of the manuscript.
